# Sonographic Risk Stratification Systems for Thyroid Nodules as Rule-Out Tests in Older Adults

**DOI:** 10.3390/cancers12092458

**Published:** 2020-08-30

**Authors:** Giorgio Grani, Gabriela Brenta, Pierpaolo Trimboli, Rosa Falcone, Valeria Ramundo, Marianna Maranghi, Piernatale Lucia, Sebastiano Filetti, Cosimo Durante

**Affiliations:** 1 Department of Translational and Precision Medicine, “Sapienza” University of Rome, Viale del Policlinico 155, 00161 Rome, Italy; giorgio.grani@uniroma1.it (G.G.); rosa.falcone@uniroma1.it (R.F.); valeria.ramundo@uniroma1.it (V.R.); marianna.maranghi@uniroma1.it (M.M.); piernatale.lucia@uniroma1.it (P.L.); 2 Endocrinology Department, Cesar Milstein Hospital, Buenos Aires CABA C1221ACI, Argentina; gbrenta@gmail.com; 3 Clinic of Endocrinology, Ente Ospedaliero Cantonale, 6900 Lugano, Switzerland; pierpaolo.trimboli@eoc.ch; 4 Faculty of Biomedical Sciences, Università della Svizzera Italiana (USI), 6900 Lugano, Switzerland

**Keywords:** ultrasonography, ultrasound, thyroid nodule, reproducibility of results, sensitivity and specificity, aged adults, elderly

## Abstract

**Simple Summary:**

The use of risk-stratification systems for thyroid nodules based on ultrasound features may reduce the number of biopsies to be performed. The aim of our study was to assess the diagnostic performance of these systems in different age groups. We confirmed that all systems had a significant discriminative performance in all age groups. The system proposed by the American College of Radiology was the best performing one, but all risk-stratification systems could avoid a sizable number of biopsies when applied as rule-out tests (to exclude malignancy) in elderly patients.

**Abstract:**

Ultrasonographic risk-stratification systems (RSS), including various Thyroid Imaging Reporting and Data Systems (TIRADS), were proposed to improve reporting and reduce the number of fine-needle aspiration biopsies. However, age might be a confounder since some suspicious ultrasonographic features lack specificity in elderly patients. We aimed to investigate whether the diagnostic performance of the RSS varied between age groups. All patients consecutively referred for thyroid biopsy between November 1, 2015, and March 10, 2020, were included. The malignancy risk of each nodule was estimated according to five RSS: the American Association of Clinical Endocrinologists/American College of Endocrinology/Associazione Medici Endocrinologi guidelines, the American College of Radiology (ACR) TIRADS, the American Thyroid Association guidelines, the European TIRADS, and the Korean TIRADS. Overall, 818 nodules (57 malignant) were evaluated. The malignancy rate was higher in patients ≤ 65 years (8.1%) than in patients > 65 years (3.8%; *p* = 0.02). All RSS confirmed a significant discriminative performance in both age groups, with a negative predictive value of 100% in patients > 65 years, although specificity was lower in older patients. The ACR TIRADS was the best performing in both age groups. RSS could avoid a sizable number of biopsies when applied as rule-out tests in elderly patients.

## 1. Introduction

Various published risk-stratification guidelines [1,2,3,4,5] provide recommendations for the evaluation of thyroid nodules based on the combination of nodule size and ultrasonographic (US) appearance [6], with the aim of improving the standardization of thyroid ultrasound reporting and the identification of the small subset of nodules that warrant fine-needle aspiration biopsy (FNAB). The performance of these systems has been validated in retrospective [7,8,9,10] and prospective studies [11,12,13] and has also been confirmed by a recent meta-analysis [14]. Classification is usually based on the recognition of patterns of sonographic features, though the American College of Radiology (ACR) Thyroid Imaging Reporting and Data System (TIRADS) [4] assigns nodules points for each of five US categories, which are then added to determine a final class. The decision of whether to perform a biopsy or monitor the nodule is based on the maximum nodule diameter, with a different threshold for each risk class. For nodules in high-risk classes, FNAB is usually indicated if the maximum diameter is 1 cm or more. For nodules in lower risk classes, the size thresholds for FNAB range from 1.5 to 3 cm, depending on the risk-stratification system. It has been demonstrated that the various risk-stratification schemes vary in their ability to reduce the number of unnecessary FNABs. However, the ACR TIRADS has been found to outperform the other risk-stratification systems in its ability to decrease the number of biopsies while improving diagnostic accuracy [7,11,14].

Most recently, the ACR TIRADS and the sonographic risk-stratification systems proposed by the American Thyroid Association (ATA) [2] and the American Association of Clinical Endocrinologists/American College of Endocrinology/Associazione Medici Endocrinologi (AACE/ACE/AME) guidelines [1] have been validated in a geriatric population [15]. In that study, it is suggested that age might be a confounder since some suspicious US features of thyroid nodules lack specificity in elderly patients [15].

The aim of this study was to investigate whether the diagnostic performance (and the number of avoided biopsies) of the five most widely used sonographic risk-stratification systems (also including the EU-TIRADS of the European Thyroid Association [3] and the K-TIRADS of the Korean Society of Thyroid Radiology [5]) varied between age groups.

## 2. Results

**Figure 1:**
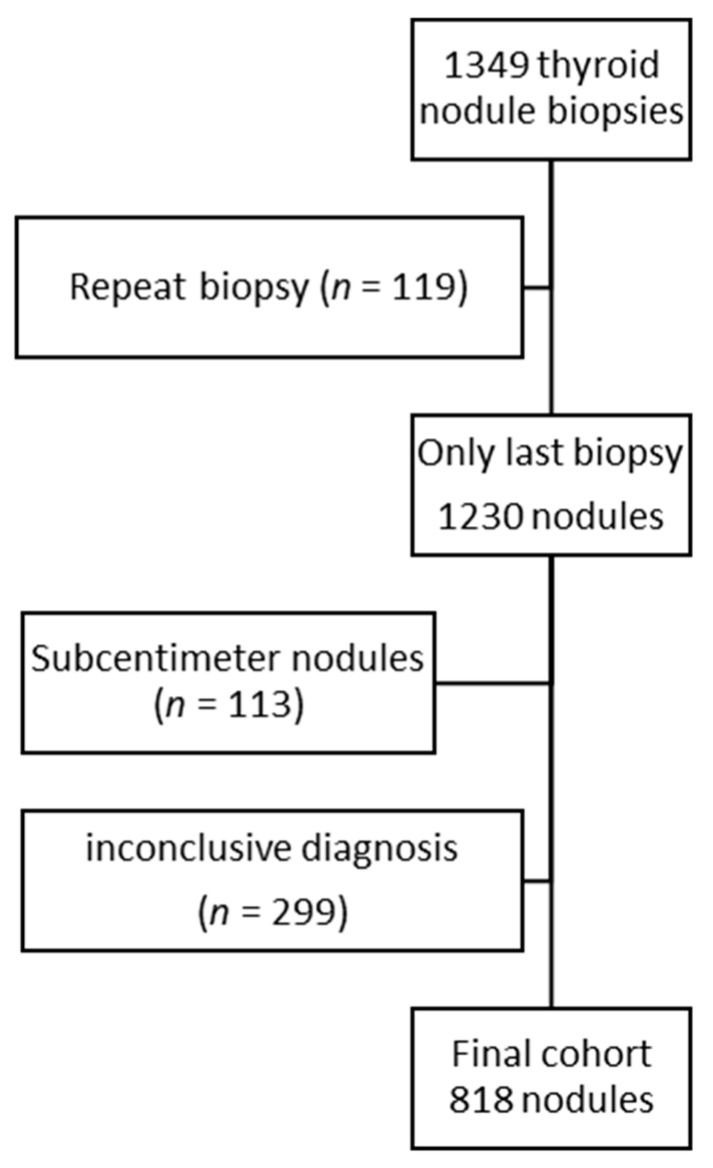
Flow chart of included nodules. The age distribution of the final cohort (median 56 years, interquartile range 46–66 years) was not significantly different from the age distribution of the excluded nodules (median 56 years, interquartile range 46–67 years; *p* = 0.75).

A total of 1349 thyroid nodule sonographic examinations before biopsy were evaluated. Some biopsies were performed multiple times on the same nodule during the study period (*n* = 119) due to cytology report suggestions, indeterminate cytology, non-diagnostic cytology, nodule growth, or the appearance of new suspicious features. In these cases, only the last examination was considered. The actual number of biopsied nodules was 1230 (1145 patients). Of these, 113 nodules were excluded because the maximum diameter was less than one centimeter, and 299 were excluded because of an inconclusive diagnosis (non-diagnostic or indeterminate cytology report without surgical pathology). To evaluate the potential impact of these exclusions on the age distribution of our final cohort, we compared the age distribution in the excluded and analyzed groups. Individuals with smaller nodules were younger (median 52 years (interquartile range, IQR 42–63) versus 57 years (IQR 47–67), *p* = 0.003), while patients with an inconclusive diagnosis were older (median 58 years (IQR 47–68 years) vs. 55 (IQR 46–66), *p* = 0.005). However, the age distribution was comparable between the group with excluded nodules and the final cohort (Figure 1).

The final cohort included 818 thyroid nodules, with a median maximum diameter of 20.7 (IQR 15–28.8) mm, of which 57 (7%) were classified as malignant. Seventy-five patients were submitted to surgery (23 benign nodules, and 52 of the malignant nodules), with a median maximum diameter of the biopsied nodule of 16.8 (IQR 13.1–27.7) mm, smaller than the not resected biopsied nodules (21.1 mm, IQR 15.4–29.1 mm; *p* = 0.025). The malignancy rate was higher in patients ≤ 65 years (8.1%) than in patients older than 65 years (3.8%; *p* = 0.02). The need for surgery was not significantly different between groups (13, 6.1% in the elderly group, and 62, 10.2% in the younger group; *p* = 0.096). We analyzed the distribution of single sonographic features (Table 1) and found no differences between the two age groups except for cystic nodules, which were more common in young patients, and calcifications, which were more frequent in the elderly. 

When using these features to classify nodules according to the five sonographic risk-stratification systems, we found no differences in the distribution of the two age groups with the AACE/ACE/AME, ACR TIRADS, and K-TIRADS systems (Table 2). However, elderly patients more commonly harbored EU-TIRADS 5 nodules and lesions that were non-classifiable in the ATA scheme (i.e., isoechoic nodules with other suspicious features like microcalcification, irregular margins, taller-than-wide shape, disrupted rim calcifications with a small extrusive hypoechoic soft tissue component, or evidence of extrathyroidal extension). However, if non-classifiable nodules were grouped with intermediate-suspicion nodules, the difference disappeared (Chi-square test; *p* = 0.214). The malignancy rate for each sonographic risk class is reported for each age group in Table 2.

Finally, we evaluated the diagnostic accuracy of the five systems by calculating sensitivity, specificity, positive and negative predictive values, and area under the receiver operating characteristic curve (AUROC) for patients younger and older than 65 years (Table 3). All systems confirmed a statistically significant discriminative performance in both age groups, with the specificity and positive predictive values of the systems being generally lower in older patients. However, all systems achieved a negative predictive value of 100% in patients > 65 years since no malignancy was missed by any of the systems. However, it is worth noting that for the ATA system, such a test performance was not confirmed if non-classifiable nodules were not submitted to biopsy. In fact, 16/172 (9.3%) of these nodules harbored a malignancy, and if they were not subjected to biopsy, the negative predictive value of the ATA system would decrease to 96.1% (95% CI 90.3–98.9%) in the > 65 group and to 94.1% (95% CI 90.4–96.6%) in patients ≤ 65 years. The application of these systems would avoid 13.2–45.3% of all FNABs in patients > 65 years. The ACR TIRADS was the best performing system as it was able to prevent the highest number of biopsies and achieve the best discriminative performance as estimated by the AUROC in both age groups.

## 3. Discussion

While the prevalence of thyroid nodules increases with increasing age, the malignancy rate is reported to be lower [16]; thus, the proper identification of the small number of lesions requiring clinical attention is of paramount importance in elderly patients. The chances of diagnosing asymptomatic thyroid nodules are increased by the frequent use of high-frequency ultrasound and cross-sectional imaging in routine clinical care [17]. However, while confirmed cancers in elderly patients are more likely to be aggressive [16], the risks associated with overtreatment of benign or low-risk malignant diseases should be carefully avoided in frail patients since the benefits are uncertain [18]. It is now clear that less aggressive treatment approaches are safe for low-risk thyroid malignancies [19,20], even if these are still relatively uncommon in real-world practice [21]. In elderly patients, an active surveillance approach may be used to defer or even definitively avoid surgery [22]. However, clinicians may be concerned by the potential occurrence of more aggressive tumors in older patients if a long-term follow-up protocol is adopted instead of immediate thyroid nodule biopsy.

In our cohort, we found that nodules submitted to biopsy in individuals > 65 years had more calcifications, even if the overall rate of malignancy was lower than in younger patients [16]. US-detected microcalcifications are associated with the presence of psammoma bodies [23] in papillary thyroid cancer. However, dystrophic or stromal calcifications and eosinophilic colloid may also appear as punctate hyperechogenic foci [24], similar to microcalcifications. 

The distribution of risk categories was comparable between age groups in the different sonographic risk-stratification systems, with the exception of the EU-TIRADS and ATA guideline systems. Due to the higher rate of microcalcifications, the number of EU-TIRADS five and ATA non-classifiable nodules was higher in older patients. The ATA non-classifiable nodules are a significant proportion of the whole cohort and have a non-negligible malignancy rate, as previously reported by other authors [25]. This is due to the presence of key suspicious features in the context of isoechogenic nodules. For this reason, as suggested in the recent literature [25], non-classifiable nodules were counted as intermediate-suspicion nodules: in this way, the difference in ATA risk category distribution between age groups disappeared. 

When analyzing the diagnostic performance of sonographic stratification systems, their discriminative ability was confirmed in people > 65 years, even if the low positive predictive values and specificities suggested the need to revise the definition or the relative weight of some features in the > 65 age group (microcalcifications seemed to be the most critical). These points should be taken into consideration when current guidelines are updated or in the development of new systems. These results were consistent with data reported by Di Fermo et al. [15], which supported the validity of sonographic stratification systems in elderly patients, even if the specificity of suspicious features, in this setting, was lower than expected. Conversely, in our cohort, there was no significant difference in diagnostic performance between the ATA and AACE/ACE/AME systems. The ACR TIRADS achieved the best discriminative performance. It is important to note that this system weighs microcalcifications and other punctate echogenic foci equally. Our results might be due to the low overall malignancy rate in our cohort. In settings with a higher pretest probability of malignancy (e.g., 18F-fluorodeoxyglucose positron emission-positive nodules), TIRADS with a higher propensity to indicate FNAB may be preferred [26].

This study had some limitations. First of all, the sample size might be limited. Most malignancies were confirmed by surgical histology, but false positives could not be excluded for patients with cytological diagnoses of malignancy who opted for conservative management. For cytologically-benign nodules, false negatives may occur, and a false negative rate of 3.7% has been reported [27]. Furthermore, we excluded subcentimeter nodules (9.2%) and lesions with inconclusive cytology (24.3%), although these exclusions did not alter the age distribution of our final cohort. The exclusion of indeterminate cytology nodules from the analysis might have reduced the amount of follicular thyroid cancers. However, scoring systems have also been found to correctly classify these cancers [28], mainly due to the suggestion to biopsy nodules greater than 20–25 mm, regardless of their sonographic pattern.

## 4. Materials and Methods 

All patients consecutively referred to our center for FNAB of a thyroid nodule between November 1, 2015, and March 10, 2020, were included in the study. The study was conducted with institutional review board approval (Sapienza University Ethics Committee, study number 806/16) and written consent. 

Patients were referred by our thyroid nodule clinic and by other specialists, including hospitalists, endocrinologists, nuclear medicine physicians, and surgeons, based on clinical risk factors, sonographic risk features, or patient preference. 

Prior to FNAB, each nodule was examined with a HI-VISION Avius^®^ system (Hitachi Medical Corporation, Inc., Tokyo, Japan) and a 13-MHz linear-array transducer. During this re-examination, two clinicians experienced in thyroid imaging recorded their joint evaluation of the sonographic features of each nodule on a standardized form. Full details on the enrollment criteria and procedures used for sonographic assessment, risk stratification, and FNAB examination of the nodules have previously been published [11,29,30]. We previously used a subset of this cohort in previous studies we conducted to compare the diagnostic performance of the systems, evaluate the impact of intrathyroidal location, and propose a better definition of the taller-than-wide shape, the results of which have already been reported [11,31,32]. In summary, all nodule sonographic features were collected, and the malignancy risk of each nodule was estimated automatically according to five sonographic risk-stratification systems by applying an algorithmic approach: the AACE/ACE/AME guidelines, the ACR TIRADS, the ATA guidelines, the EU-TIRADS, and the K-TIRADS. Nodules that could not be classified with the ATA guidelines were considered intermediate-suspicion nodules (i.e., iso or hyperechoic nodules with high-suspicion features, including irregular margins, microcalcifications, taller-than-wide shape, disrupted rim calcifications with a small extrusive hypoechoic soft tissue component, or evidence of extrathyroidal extension) [25]. Nodules with a maximum diameter of less than 1 cm were excluded from this study since none of the risk-stratification systems routinely recommend FNAB for subcentimeter thyroid nodules. 

### 4.1. Reference Standard

Cytology was classified according to the criteria published in the Italian consensus for thyroid cytopathology [33,34], a six-tiered system comparable to the Bethesda System for Reporting Thyroid Cytopathology. If surgery had been performed, the reference standard diagnosis (malignant vs. benign) was based on histological examination of the resected nodule. If the nodule was not resected, a cytology-based reference standard was applied. Nodules were considered malignant if they were classified as TIR4 or TIR5 (corresponding to Bethesda classes V and VI), and benign if they were classified as TIR2, corresponding to Bethesda class II. Unresected nodules that were cytologically classified as TIR1 (non-diagnostic), 3A (low-risk indeterminate), or 3B (high-risk indeterminate) were excluded. 

### 4.2. Age Groups

Patients were grouped according to their chronological age, a younger group (≤65 years), and an elderly group (>65 years). It is the classical, conventional threshold, that we adopted, even if it is subject to changes based on comprehensive evidence in various aspects of social, cultural, and medical sciences [35]. 

### 4.3. Statistical Analysis

The nodules for which FNAB was indicated in each system were flagged as test positive. The sensitivity, specificity, positive and negative predictive values (PPV and NPV), and the AUROC, each with 95% confidence intervals, were computed for each system. Differences in categorical variables between groups were analyzed using the Chi-square test or the Fisher exact test. 

The proportion of biopsies that would not have been indicated by the various systems were compared using the McNemar test. Data were analyzed with IBM SPSS Statistics, version 25.0 (IBM Corp., Armonk, NY, US). AUROC was compared with the DeLong approach [36] using the easyROC package [37].

## 5. Conclusions

In conclusion, when current risk-stratification systems were applied in clinical practice as rule-out tests for older patients, all were able to avoid a sizable number of biopsies, with a negative predictive value of 100%. Indeed, no malignancy was missed in any of the systems, though this result required that non-classifiable nodules in the ATA guidelines be considered intermediate-suspicion lesions. As previously reported in the general population, the ACR TIRADS outperformed the other systems as it avoided the highest number of biopsies and had the best discriminative power in the > 65 age group.

## Figures and Tables

**Table 1:** Sonographic features of thyroid nodules according to age group.

Feature	Descriptor	Age		Total	*p*-Value ^1^
		≤65 years	>65 years		
**Maximum diameter, mm (IQR)**		21 (14.9–29.2)	20.4 (15.2–27.7)	20.7 (15–28.8)	0.798 ^2^
**Margins**	Regular	245	68	313	0.246
		40.4%	32.1%	38.3%	
	Irregular/lobulated	92	32	124	
		15.2%	15.1%	15.2%	
	Ill-defined	37	14	51	
		6.1%	6.6%	6.2%	
	Infiltrating	3	1	4	
		0.5%	0.5%	0.5%	
	Hypoechoic halo	229	97	326	
		37.8%	45.8%	39.9%	
**Cystic composition**		23	1	24	**0.006**
		3.8%	0.5%	2.9%	
**Solid composition**		180	66	246	0.379
		29.7%	31.1%	30.1%	
**Mixed composition**	Septa	20	7	27	0.271
		3.3%	3.3%	3.3%	
	Non-nodular	364	137	501	
		60.1%	64.6%	61.2%	
	Central nodular solid portion	6	1	7	
		1%	0.5%	1%	
	Eccentric nodular solid portion	13	0	13	
		2.1%	0%	1.6%	
**Echogenicity**	Anechogenic	7	1	8	0.107
		1.2%	0.5%	1.0%	
	Hyperechogenic	4	3	7	
		0.7%	1.4%	0.9%	
	Isoechogenic	427	167	594	
		70.5%	78.8%	72.6%	
	Hypoechogenic	147	36	183	
		24.3%	17.0%	22.4%	
	Markedly hypoechogenic	21	5	26	
		3.5%	2.4%	3.2%	
**Hyperechoic Foci**	None	437	162	599	0.277
		72.1%	76.4%	73.2%	
	Comet-tail	43	9	52	
		7.1%	4.2%	6.4%	
	Indeterminate	126	41	167	
		20.8%	19.3%	20.4%	
**Calcifications**	None	486	147	633	**0.005**
		80.2%	69.3%	77.4%	
	Macrocalcifications	74	42	116	
		12.2%	19.8%	14.2%	
	Microcalcifications	46	23	69	
		7.6%	10.8%	8.4%	
**Suspicious extrathyroidal extension**		6	1	7	0.422
		1.0%	0.5%	0.9%	
**Suspicious lymph nodes**		10	2	12	0.361
		1.7%	0.9%	1.5%	
**Taller-than-wide shape**		101	42	143	0.175
		16.7%	19.8%	17.5%	
**Total**		606	212	818	

^1^ Differences in the distribution of sonographic features between age groups were analyzed using the Chi-square test or the Fisher exact test. ^2^ Differences in size between age groups were analyzed using the Mann–Whitney U test. *p*-values < 0.05 are highlighted in bold.

**Table 2:** Distribution of risk classes according to 5 sonographic classification systems and actual malignancy rate in the two age groups.

RSS	Category	Age		Total	*p*-Value ^1^	Malignancy Rate		
		≤65 years	>65 years			≤65 years	>65 years	Overall
**ATA guidelines**	Benign	6	0	6	**0.041**	0	-	0
		1.0%	0%	0.7%		0%	-	0%
	Very low suspicion	301	94	395		5	0	5
		49.7%	44.3%	48.3%		1.7%	0%	1.3%
	Low suspicion	78	33	111		1	1	2
		12.9%	15.6%	13.6%		1.3%	3.0%	1.8%
	Intermediate suspicion	31	5	36		6	0	6
		5.1%	2.4%	4.4%		19.4%	0%	16.7%
	High suspicion	75	23	98		25	3	28
		12.4%	10.8%	12.0%		33.3%	13.0%	28.6%
	Not classifiable	115	57	172		12	4	16
		**19.0%**	**26.9%**	**21.0%**		10.4%	7.0%	9.3%
**K-TIRADS**	K-TIRADS 2	11	2	13	0.477	0	0	0
		1.8%	0.9%	1.6%		0%	0%	0%
	K-TIRADS 3	375	125	500		7	1	8
		61.9%	59.0%	61.1%		1.9%	0.8%	1.6%
	K-TIRADS 4	179	73	252		20	6	26
		29.5%	34.4%	30.8%		11.2%	8.2%	10.3%
	K-TIRADS 5	41	12	53		22	1	23
		6.8%	5.7%	6.5%		53.7%	8.3%	43.4%
**AACE/ACE/AME**	Low risk	48	11	59	0.190	0	0	0
		7.9%	5.2%	7.2%		0%	0%	0%
	Intermediate risk	358	119	477		10	1	11
		59.1%	56.1%	58.3%		2.8%	0.8%	2.3%
	High risk	200	82	282		39	7	46
		33.0%	38.7%	34.5%		19.5%	8.5%	16.3%
**ACR TIRADS**	TR1	24	5	29	0.489	0	0	0
		4.0%	2.4%	3.5%		0%	0%	0%
	TR2	164	48	212		2	0	2
		27.1%	22.6%	25.9%		1.2%	0%	0.9%
	TR3	106	39	145		2	0	2
		17.5%	18.4%	17.7%		1.9%	0%	1.4%
	TR4	208	83	291		13	5	18
		34.3%	39.2%	35.6%		6.3%	6.0%	6.2%
	TR5	104	37	141		32	3	35
		17.2%	17.5%	17.2%		30.8%	8.1%	24.8%
**EU-TIRADS**	EU TIRADS 2	6	1	7	**0.035**	0	0	0
		1.0%	0.5%	0.9%		0%	0%	0%
	EU TIRADS 3	318	113	431		6	1	7
		52.5%	53.3%	52.7%		1.9%	0.9%	1.6%
	EU TIRADS 4	88	16	104		6	0	6
		14.5%	7.5%	12.7%		6.8%	0%	5.8%
	EU TIRADS 5	194	82	276		37	7	44
		32.0%	38.7%	33.7%		19.1%	8.5%	15.9%

Abbreviations: AACE/ACE/AME: American Association of Clinical Endocrinologists/American College of Endocrinology/Associazione Medici Endocrinologi; ACR TIRADS: American College of Radiology Thyroid Imaging Reporting and Data System; ATA: American Thyroid Association; EU-TIRADS: European Thyroid Imaging Reporting and Data System; K-TIRADS: Korean Thyroid Imaging Reporting and Data System; RSS: sonographic risk-stratification system. ^1^ Differences in the distribution of RSS classes between age groups were analyzed using the Chi-square test. *p*-values <0.05 are highlighted in bold.

**Table 3:** Diagnostic performance of the 5 sonographic stratification systems, stratified according to age ≤ 65 years or > 65 years.

RSS		Avoided Biopsies (%) ^b^	Sensitivity (95% CI)	Specificity (95% CI)	PPV (95% CI)	NPV (95% CI)	AUROC (95% CI) ^c^
**≤65 years**	ACR TIRADS	305	83.67%	53.3%	13.6%	97.4%	0.68
		(50.3%)	(70.3–92.68%)	(49.1–57.5%)	(10.0–18.0%)	(94.9–98.9%)	(0.63–0.74)
	AACE/ACE/AME	197 *	87.8%	34.3%	10.5%	96.95%	0.61
		(32.5%)	(75.2–95.4%)	(30.3–38.4%)	(7.7–13.9%)	(93.5–98.9%)	(0.56–0.66)
	ATA ^a^	137 *	93.9%	24.1%	9.8%	97.8%	0.59
		(22.6%)	(83.1–98.7%)	(20.6–27.8%)	(7.3–12.9%)	(93.7–99.5%)	(0.55–0.63)
	EU-TIRADS	154 *	85.7%	26.4%	9.3%	95.4%	0.56
		(25.4%)	(72.8–94.1%)	(22.8–30.3%)	(6.8–12.3%)	(90.9–98.15%)	(0.51–0.61)
	K-TIRADS	79 *	95.9%	13.8%	8.9%	97.47%	0.55
		(13.0%)	(86.0–99.5%)	(11.1–17%)	(6.6–11.7%)	(91.1–99.7%)	(0.52–0.58)
**>65 years**	ACR TIRADS	96	100.0%	47.1%	6.9%	100.0%	0.73
		(45.3%)	(63.1–100.0%)	(40.0–54.1%)	(3.0–13.1%)	(96.2–100.0%)	(0.70–0.77)
	AACE/ACE/AME	61 ^#^	100.0%	29.9%	5.3%	100.0%	0.65
		(28.8%)	(63.1–100.0%)	(23.7–36.7%)	(2.3–10.2%)	(94.1–100.0%)	(0.62–0.68)
	ATA ^a^	46 ^#^	100.0%	22.5%	4.8%	100.0%	0.61
		(21.7%)	(63.1–100.0%)	(17.0–28.9%)	(2.1–9.3%)	(92.3–100.0%)	(0.58–0.64)
	EU-TIRADS	52 ^#^	100.0%	25.5%	5.0%	100.0%	0.63
		(24.5%)	(63.1–100.0%)	(19.7–32.0%)	(2.2–9.6%)	(93.1–100.0%)	(0.60–0.66)
	K-TIRADS	28 ^#^	100.0%	13.7%	4.3%	100.0%	0.57
		(13.2%)	(63.1–100.0%)	(9.3–19.2%)	(1.9–8.4%)	(87.7–100.0%)	(0.54–0.59)

Abbreviations: AUROC: area under the receiver operating characteristic curve; CI: confidence interval; NPV: negative predictive value; PPV: positive predictive value. a) nodules not classifiable with the ATA system were considered intermediate-suspicion nodules. b) comparison with ACR TIRADS in the ≤ 65 age group, McNemar test, * *p* < 0.001; comparison with ACR TIRADS in the > 65 age group, McNemar test, # *p* < 0.001. c) comparison with ACR TIRADS in the ≤ 65 age group, DeLong approach, ACR vs. ATA: *p* = 0.006; ACR vs. AACE: *p* = 0.05; ACR vs. K-TIRADS: *p* < 0.001; ACR vs. EU-TIRADS: *p* = 0.002; AACE vs. K-TIRADS: *p* = 0.04; comparison with ACR TIRADS in the > 65 age group, DeLong approach, ACR vs. all other systems: *p* < 0.001; AACE vs. K-TIRADS: *p* < 0.001; EU-TIRADS vs. K-TIRADS: *p* = 0.002.
